# Subjective Signs of Rhegmatogenous Retinal Detachment Associated With Acute Posterior Vitreous Detachment

**DOI:** 10.4021/jocmr1243w

**Published:** 2013-04-23

**Authors:** Ken-ichi Sato, Shin-ichi Nishimura

**Affiliations:** a Department of Ophthalmology, Nikko Memorial Hospital, 1-5-13 Shintomi-cho, Muroran, Hokkaido, Japan

## To the Editor

Despite recent anatomical successes of surgical treatment for rhegmatogenous retinal detachment (RRD), reattachment fails in 1.1-2.5% of cases [1-3]. In addition, even in cases of reattachment, some patients continue to show poor functional results [1, 2]. Prevention of RRD is therefore undoubtedly desirable. We retrospectively examined subjective signs in patients who underwent surgery for RRD to determine whether they could have received prophylactic laser treatment [4] at an early stage of retinal tear only or minimal retinal detachment.

Subjects were 34 consecutive patients (34 eyes; 10 females; mean age ± SD: 58 ± 11 years) who presented with RRD associated with acute posterior vitreous detachment (PVD) on initial visit to Nikko Memorial Hospital. All patients subsequently underwent pars plana vitrectomy between April 2009 and March 2012; 3 were excluded due to complications related to obvious vitreous hemorrhage. Cases with traumatic or atopic RRD were not included. At the initial visit, patients were interviewed about subjective visual symptoms.

Informed consent was obtained from all subjects prior to surgery and the study adhered to the tenets of the Declaration of Helsinki.

**Figure 1:**
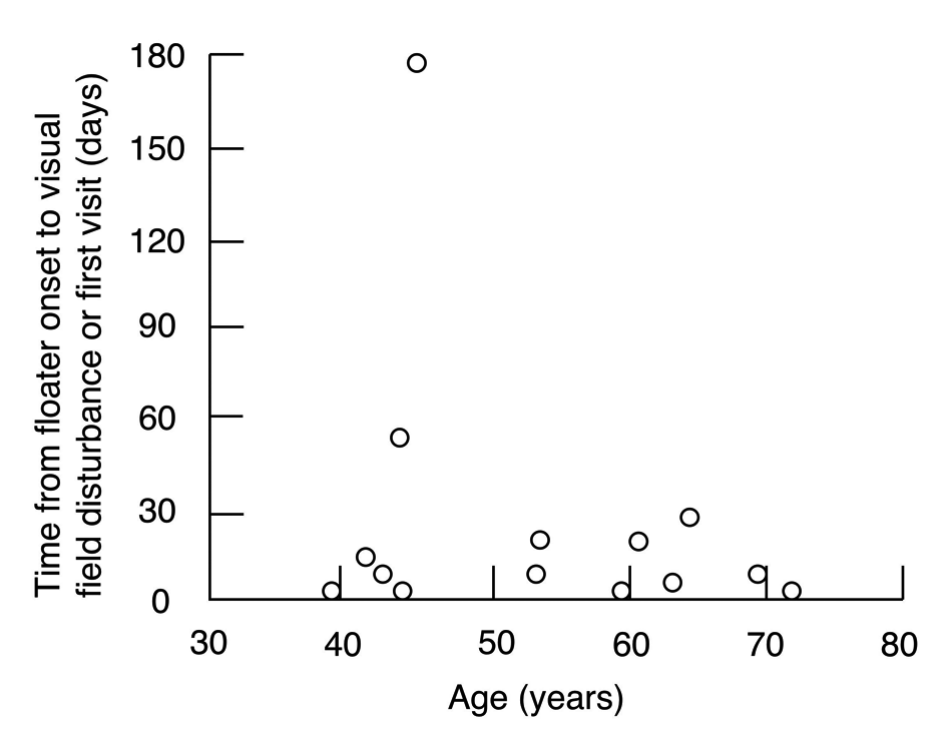
Correlation between age and time from onset of floaters to experiencing visual field disturbance.

Fourteen patients (41%) visited the hospital because of floaters only or visual field disturbance following floaters; the remainder visited due to visual field disturbance, presumably caused by RRD, with no experience of floaters. Only one patient complained of photopsia with initial floaters. Of those who initially experienced floaters, 2 patients took more than one month from onset to experiencing visual field disturbance (Fig. 1); this late-visit group was significantly younger than the other patients (P = 0.007, Welch’s t test).

A significant proportion of patients with acute PVD complain of monocular floaters [5]. In the present study, we therefore considered the onset of floaters to be a potential sign of acute PVD. Patients also showing apparent vitreous hemorrhage on initial visit were excluded from the study because associated floaters may bias the patients’ actions in seeking a consultation.

Of the patients without preceding floaters, approximately 60% visited due to visual field disturbance caused by RRD. As a result, it was too late to perform prophylactic laser treatment by the time the subjective symptoms led to consultation.

In some of the relatively young patients with preceding floaters, there was quite a long period of time from onset of the initial floater to experiencing visual field disturbance. Since vitreous syneresis has yet to advance in the young generally [6], the tamponade effect of gel-state vitreous or weak vitreous contractions may result in late-onset RRD. Thus, consultation immediately after the onset of floaters might enable prophylactic laser photocoagulation to be performed at an early stage when only retinal tear or minimal retinal detachment has occurred. This suggests the importance of awareness of this state among the relatively young.

Several studies suggest that patients with symptomatic PVD do not necessarily need scheduled reexamination if there are no pigmented cells in the vitreous, vitreous hemorrhage, or retinal hemorrhage at initial fundus examination [7-9]. Nevertheless, considering the potential for late-onset retinal breaks or RRD, it may be worth patients younger than 50 to schedule a follow-up examination after initial consultation.

The study had certain limitations. This was a single-center study and there were a limited number of patients. A larger scale study is warranted to verify the results.
